# An efficient, accurate and clinically-applicable index of content word fluency in Aphasia

**DOI:** 10.1080/02687038.2021.1923946

**Published:** 2021-06-25

**Authors:** Reem S. W. Alyahya, Paul Conroy, Ajay D. Halai, Matthew A. Lambon Ralph

**Affiliations:** a Communication and Swallowing Disorders Department, King Fahad Medical City, Riyadh, Saudi Arabia; b MRC Cognition and Brain Sciences Unit, University of Cambridge, Cambridge, UK; c College of Medicine, Alfaisal University, Riyadh, Saudi Arabia; d Division of Neuroscience and Experimental Psychology, University of Manchester, Manchester, UK

**Keywords:** Aphasia, connected speech, discourse, fluency, word retrieval

## Abstract

**Background:**

Despite the clinical importance of assessing the efficiency and accuracy of fluency in terms of content words production during connected speech, assessments based on discourse tasks are very time-consuming and thus not clinically feasible.

**Aims:**

(1) Examine the relationship between single-word naming and word retrieval during discourse production. (2) Investigate the relationship between word retrieval and content word fluency derived from a simple versus naturalistic discourse tasks. (3) Develop and validate an efficient and accurate index of content word fluency that is clinically viable.

**Methods:**

Two discourse tasks (simple picture description and naturalistic storytelling narrative) were collected from 46 participants with post-stroke aphasia, and 20 age/education matched neuro-typical controls. Each discourse sample was fully transcribed and quantitative analysis was applied to each sample to measure word retrieval and content word fluency. Three single-word naming tasks were also administered to each participant with aphasia.

**Results:**

Correlational analyses between single-word naming and word retrieval in connected speech revealed weak/moderate relationships. Conversely, strong correlations were found between measures derived from simple picture description against naturalistic storytelling discourse tasks. Moreover, we derived a novel, transcription-less index of content word fluency from the discourse samples of an independent group (neuro-typical controls), and then we validated this index across two discourse tasks in the tested group (persons with aphasia). Correlation and regression analyses revealed extremely strong relationships between participants’ (neuro-typical controls and persons with aphasia) scores on the novel index and measures of content word fluency derived from the formal transcription and quantitative analyses of discourse samples, indicating high accuracy and validity of the new index.

**Conclusions:**

Simple picture description rather than picture naming provides a better estimate of word retrieval in naturalistic connected speech. The novel developed index is transcription-less and can be implemented online to provide an accurate and efficient measure of content word fluency. Thus, it is viable during clinical practice for assessment purposes, and possibly as an outcome measure to monitor therapy effectiveness, which can also be used in randomised clinical trials.

## Introduction

Conversation between people relies heavily on producing fluent-connected speech. The cornerstone of informativeness in connected speech is content word production and retrieval. Producing contentful connected speech is invariably impaired to some degree in people with acquired language impairments (i.e., aphasia) following brain damage or neurological disorders. Aphasia has a remarkable impact on fluency and the production of connected speech. Given its centrality to expressive language and high prevalence of impairment, it is essential to secure fast, efficient and accurate indexes of content word fluency for clinical practice, research and as potential outcome measures in randomized control trials.

Word retrieval is commonly assessed in persons with aphasia (PWA) using picture naming, a clinically efficient measure of single-word retrieval, which is an essential task in all widely used aphasia assessment batteries: the Boston Diagnostic Aphasia Examination (BDAE: Goodglass & Kaplan, 1983), the Comprehensive Aphasia Test (CAT: Swinburn et al., 2005), and the Western Aphasia Battery (WAB: Kertesz, 1982). The existing aphasiological literature indicates that word retrieval at the single-word naming level does not correlate or predict expressive performance on connected speech at discourse levels, as studies found no correlations between word retrieval during picture naming and picture description or conversational speech (Basso et al., 1990; Bastiaanse & Jonkers, 1998). These studies, however, were challenged by other studies that reported high positive correlations between word retrieval of single nouns (Herbert et al., 2008) and verbs (Mayer & Murray, 2003; Williams & Canter, 1987) against composite picture description or conversational speech tasks. We note, however, that the correlations were not significant when analysing data from people with mild versus moderate aphasia separately (Mayer & Murray, 2003), and the strength of correlation varied across different aphasia classifications (Williams & Canter, 1987). Another study showed that word retrieval was more impaired for single-word tasks compared to narratives in people with mild anomic aphasia (Pashek & Tompkins, 2002). It must be noted that small samples of PWA were included in these studies. A more recent study modelled confrontation naming and the proportion of paraphasias in a large group of PWA found naming test scores not to be a strong predictor of performance in connected speech (Fergadiotis & Wright, 2016).

Moving beyond single-word naming tasks, picture description is also commonly used in clinical examination, and it is a subtest in all major aphasia assessment batteries: the BDAE (Goodglass & Kaplan, 1983), the CAT (Swinburn et al., 2005) and the WAB (Kertesz, 1982). This simple task elicits a short discourse by way of describing a static scene. A more extensive, naturalistic form of connected speech is storytelling narratives, which are richer not only in terms of length (Alyahya et al., 2020) but they usually involve a greater array of participants, actions and events (including spatial and temporal shifts). Thus, storytelling narratives can elicit a greater quantity and diversity of content words (Alyahya et al., 2020; Fergadiotis & Wright, 2011), more propositional density (Stark, 2019) and a wider range of word frequency and imageability (Alyahya et al., 2018c) compared to picture description in neuro-typical adults and PWA.

In the aphasiological literature, discourse production has provided rich data on fluency and insights on various linguistic elements of connected speech (Bryant et al., 2016; Dipper & Pritchard, 2017). However, there is no consensus on which discourse measures to select that can accurately reflect word retrieval or fluency in terms of content words production during connected speech, as highlighted in a comprehensive review of studies that assessed expressive language in aphasia using discourse analysis (Bryant et al., 2016). The authors of this review argued that choosing appropriate discourse measures to assess different elements of expressive language is highly challenged by the wealth of measures used in the aphasiology discourse literature, in which they found 536 different linguistic measures (e.g., words-per-minute, proportional density, word counts, and correct information unit) applied in different studies (Bryant et al., 2016). The authors also argued that this has a major impact on clinical translation of the aphasiology discourse research. Furthermore, discourse samples are very time-consuming and effortful to collect, transcribe and analyse, and because they are typically done offline, their utilisation is impractical in clinical settings. A survey showed that only 5% of Speech-Language Pathologists/Therapists use discourse tasks in their clinical examination (Simmons-Mackie et al., 2005). A review indicated that time constraint was the main barrier (Bryant et al., 2016). Depending on aphasia severity and the amount of discourse analysis, it has been estimated that every minute of discourse could take up to one full hour for transcription and analysis (Armstrong et al., 2007). The need to develop fast and efficient approaches to analyse discourse responses, ideally without the need for transcription to be used in clinical practice (Armstrong et al., 2007; Kim et al., 2019), and as part of a core outcome set of discourse measures (Dietz & Boyle, 2018) has been highlighted.

To overcome these challenges, lexicon-based analysis of discourse samples (referred to as target lexicon or core-lexicon), that does not require transcription, has been introduced (Dalton & Richardson, 2015; MacWhinney et al., 2010) and developed (Dalton et al., 2020; Kim et al., 2019). The goal is to create a checklist of lexical items in a specific discourse task and use it to examine whether the speaker has these lexical items in their active vocabulary as a measure of word retrieval in a quick way. Thus, the scoring involves assigning one point to each item, regardless of how often the word is produced. Core-lexicon lists have been mainly developed using discourse tasks from the AphasiaBank database (Dalton et al., 2020; Dalton & Richardson, 2015; MacWhinney et al., 2010). The findings from these studies have indicated that the use of these lists can differentiate the performance of PWA from healthy controls, and between different aphasia classifications (Dalton et al., 2020; Dalton & Richardson, 2015). Findings from these studies are promising by providing a measure of word retrieval, which appears to correlate with another discourse measure (i.e., main concept analysis: Dalton & Richardson, 2015) and relates to scores on a standardised aphasia test, where a verb list – but not noun, adjective or adverb lists – appeared to be sensitive to aphasia severity as measured by the WAB (Kim et al., 2019).

In the current study, we altered the standard procedure used in core-lexicon to address a different goal, which is to provide a quick estimate of the number of content words the speaker can produce in a given discourse (i.e., develop an index of content word fluency). Hence, a different and novel scoring system is created in this study to count each time a content word is produced during discourse to reflect content word fluency rather than unique word retrieval as done in previous studies. Additionally, we used a valid, data-driven approach to create the lists rather than choosing an arbitrary cut-off to include the top 25 words in each list as done previously (Kim et al., 2019; MacWhinney et al., 2010). One way to do this is to derive a target-checklist from the discourse responses of a group of neuro-typical controls and include the most frequently used items, i.e., words produced by the majority of controls. This approach appears to be appropriate given that PWA attempts to access words from the full lexical-semantic space that is utilised by neuro-typical controls, albeit at a reduced rate (Alyahya et al., 2018c). Furthermore, it has been highlighted that accuracy on the transcription-less index must be compared to measures derived from the fully transcribed samples (Dalton et al., 2020), and indeed the correlation must be high and strong for the index to be deemed sensitive and clinically valid. Therefore, in this study, we validate the sensitivity of the newly developed index by comparing the performance of this index to the performance derived using the conventional full transcription and quantitative analysis of discourse samples. This was initially done using a dataset from a group of neuro-typical controls, and then validated across a group of PWA. To provide further validation, this was done across two different discourse tasks in both groups. This forms the development of an efficient yet sensitive index of content word fluency.

We addressed three aims in this study. First, we systematically examined the relationship between the accuracy of word retrieval during single-word naming against word retrieval measures extracted from discourse samples. Discourse samples included two tasks: a simple picture description task (commonly used in clinical settings), and a storytelling narrative task (representing a naturalistic form of connected speech production). Second, we examined the relationship between word retrieval and content word fluency across the two discourse tasks, and the relationship between these discourse measures and a wide range of language and cognitive tests that assesses repetition, naming, comprehension, memory and abstract thinking. This was done to determine whether word retrieval and/or content word fluency during connected speech could be predicted by other language and cognitive tests. The third and main aim of this study was addressed due to the complexity and time involved in the standard processing and scoring of discourse samples. Specifically, we developed a novel and time-efficient index of content word fluency, derived from a transcription-less target checklist. This new index can be administered online with minimal effort, making its application clinically viable. In order to evaluate the validity of the new index, accuracy of this index was compared to measures of content word fluency in connected speech extracted from the formal transcription and quantitative analysis of discourse samples. These aims were addressed across a large group of people representing a wide range of chronic post-stroke aphasia severity/classification (spanning from mild anomia to global aphasia).

## Methods

### Participants

Forty-six participants who had developed aphasia following a single left hemorrhagic or ischemic stroke participated in this study. The BDAE (Goodglass & Kaplan, 1983) was administered to each participant, and they were diagnosed and classified according to the BDAE standard aphasia classification criteria. They were >12 months post-stroke, right-handed, native English-speakers with normal or corrected-to-normal vision and/or hearing. The exclusion criteria included multiple strokes, any other neurological conditions, severe motor-speech disorders, or being pre-morbidly left-handed. No restrictions were placed on aphasia severity or classification, in order to sample the full range of aphasia. Discourse samples were also collected from 20, age-/education-matched neuro-typical adults (two-tail *p* > 0.05 between the neuro-typical group and the PWA group on their age and education level). They were native English-speakers, right-handed, and they reported no abnormal neurological conditions or history of brain injury. Demographic information for both groups is presented in Table 1. Informed consent was obtained from all participants prior to participation under approval from local ethics committee.

### Discourse elicitation, transcription and coding

Two discourse elicitation tasks were employed to evoke simple and naturalistic monologue discourse samples. First, a simple and commonly used task was administered using the “Cookie Theft” composite picture description from the BDAE (Goodglass & Kaplan, 1983). Participants were asked to describe what is going on in the picture. Second, a naturalistic discourse response was elicited using the “Dinner Party” pictorial script of a series of eight black-and-white cartoons (Fletcher & Birt, 1983). Storytelling tasks can be richer than picture description in terms of quantity and diversity of content words in both PWA and neuro-typical adults (Alyahya et al., 2020; Fergadiotis & Wright, 2011; Stark, 2019), and responses can be twice as long as the ones elicited using the “Cookie Theft” (Alyahya et al., 2020). Participants were presented with a full storyboard for them to look through first and then asked to narrate the story. In both tasks, picture stimuli were kept in place and there was no time limit imposed during responses. No prompts or questions were provided by the examiner throughout testing, except nonverbal encouragements.

Each discourse sample was digitally recorded and then transcribed verbatim (ortho-graphically not phonetically) and checked against the recording to correct for any discrepancies, followed by content analysis conducted by the first author (RSWA), a qualified and experienced Speech-Language Pathologist. We set a high threshold for words to be sufficiently clear to be transcribed as real words, and errors were transcribed as they were heard in word form. Two measures were extracted from each transcript. First, as a measure of content word fluency, we used the correct information units (CIU) (Nicholas & Brookshire, 1993). This is a sum of all intelligible and relevant words, including words in incomplete utterances and those used in a grammatically incorrect form, and can be nouns, verbs, adjectives, adverbs, pronouns, conjunctions, articles, prepositions, numerals, and possessives; but excluding immediate repetition or perseverations of the same word or utterance. Contractions (e.g., it’s or haven’t) were counted as two separate words. Second, as a measure of word retrieval, the number of different words (NDW) (Miller, 1991) was collated.

### Naming tests

Single-word retrieval was assessed using three picture naming tests: the Boston Naming Test (BNT) (Kaplan et al., 1983), the 64-item Cambridge Naming Test (CNT) (Bozeat et al., 2000), and action pictures from the Object and Action Naming Battery (ANB) (Druks & Masterson, 2000). Each picture was presented for 10 seconds, and participants were instructed to name it aloud using a single word, and no further cues were provided. Only the first intelligible response was entered into the accuracy analysis.

### Language and cognitive assessments

In addition to picture naming and discourse samples, a range of language and cognitive tests were utilised to determine whether any of these tests would relate to word retrieval and/or content word fluency during connected speech. This includes: (i) comprehension tests: spoken word-to-picture matching test (Bozeat et al., 2000); noun picture-to-word matching (Alyahya, Halai, Conroy & Lambon Ralph, 2018b), verb picture-to-word matching (Alyahya et al., 2018a), and spoken sentence comprehension (Swinburn et al., 2005); (ii) immediate word repetition (Kay et al., 1992); (iii) memory tests: forward and backward digit span (Wechsler, 1987); and (iv) executive tests: Brixton Spatial Rule Anticipation (Burgess & Shallice, 1997), and Raven’s Coloured Progressive Matrices (Raven, 1962). Participants’ performance on naming tests and these assessments is provided in Appendix A.

### Statistical analyses

Initially, we examined the correlations between naming accuracy on three naming tests (BNT, CNT, and ANB), and word retrieval during discourse production (i.e., NDW extracted from picture description and storytelling discourse tasks) using Pearson’s correlation coefficient (Bonferroni corrected alpha of *p* < 0.016).

Second, we examined the relationship between word retrieval and content word fluency across the two discourse tasks using Pearson’s correlation coefficient analyses using CIU and NDW extracted from picture description against those extracted from storytelling narrative. Furthermore, a simultaneous multiple-regression analysis was conducted once on CIU and again on NDW produced during storytelling narrative to determine which of the naming tests, other language, and cognitive tests, or similar measures derived from picture description relate to word retrieval and content word fluency during naturalistic discourse production.

Third, we examined the discourse samples for a sensitive yet time-efficient index of content word fluency during connected speech. In order to provide an estimate of out-of-sample prediction accuracy, we designed and tested the index using the neuro-typical control group’s dataset – an entirely separate dataset from the PWA’s dataset – and then tested and validated this index in the tested group (PWA). Specifically, we derived a target-checklist from an independent group of neuro-typical controls reflecting the content words most commonly produced during “Cookie Theft” picture description samples by rank ordering the produced words in terms of frequency, and took the most commonly/consistently produced content words by the majority (i.e., ≥75%) of the neuro-typical controls irrespective of the word class. A cumulative score on the target-checklist was computed (i.e., every time the participant produced one of the target words, the total count increased, including when the target words were used again in subsequent phrases). This was done in order to provide an index of content word fluency rather than word retrieval (which was done in previous studies as indicated in the Introduction). To ensure high ecological validity of this approach, scores using this novel measure must highly correlate with the standard approach used to measure content word fluency. Therefore, Pearson’s correlation coefficient was conducted to determine the similarity between scores on the simplified target checklist and CIU extracted following transcribing the discourse samples. This approach was then applied to the dataset using PWA. Scores of this new index were computed on the picture description samples by PWA, followed by Pearson’s correlation coefficient analysis.

To further validate this new approach, the target-checklist and correlation analysis was applied on the “Dinner Party” storytelling narrative, in which the checklist was designed and tested using the neuro-typical control’s discourse samples, and then applied on the PWA’s discourse samples. Finally, to identify how content word fluency can be indexed using the new approach, simple linear regression models were created on CIU extracted from the transcripts of both picture description and storytelling samples by PWA using scores on the new content word fluency index.

## Results

Descriptive statistics on the measures extracted from the discourse samples, including length and duration, by PWA and neuro-typical controls are provided in Table 2.

### A) The relationship between picture naming and word retrieval during discourse production

**Figure 1:**
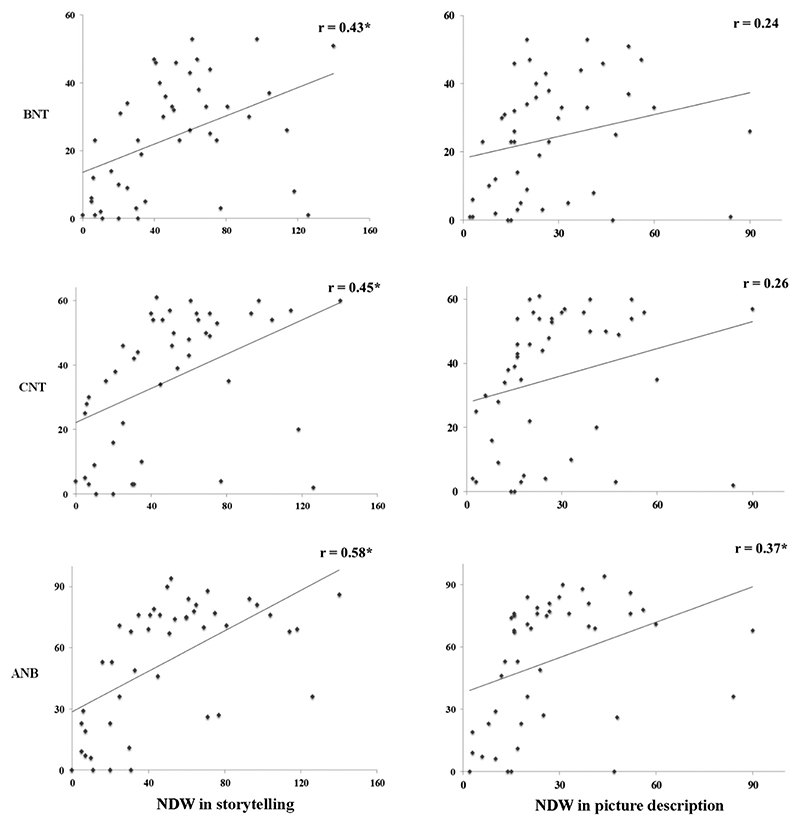
Correlations between word retrieval during picture naming and discourse production in post-stroke aphasia. Scatterplots illustrating the correlations between picture naming accuracy (y-axis) and word retrieval during two discourse tasks measured using NDW (x-axis). Significant correlations (p < 0.01) are indicated with an asterisk. BNT = Boston Naming Test (Kaplan et al., 1983), CNT = 64-item Cambridge Naming Test (Bozeat et al., 2000), ANB = action pictures from the Object and Action Naming Battery (Druks & Masterson, 2000).

Results from Pearson’s correlation coefficient analyses (Figure 1) revealed: (i) a significant albeit weak positive correlation between NDW during picture description and naming accuracy on ANB (*r* = 0.37) only, and the correlations were not significant on the other naming tests (BNT and CNT); and (ii) significant moderate positive correlations between NDW during storytelling narrative and naming accuracy on BNT (*r* = 0.43), CNT (*r* = 0.45), and ANB (*r* = 0.58). These results indicate that picture naming provides at best a moderate estimate of word retrieval during connected speech production.

### B) The relationship between word retrieval/content word fluency during discourse production and other language and cognitive tests

**Figure 2:**
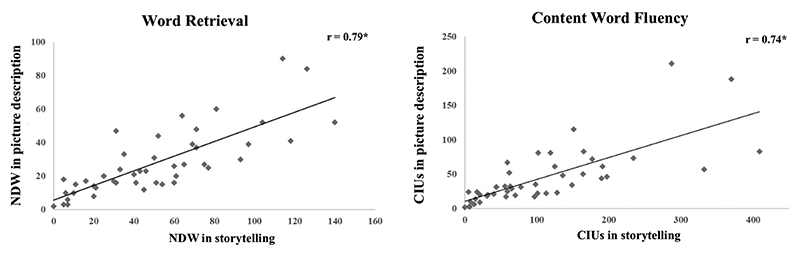
Correlations of word retrieval and content word fluency between different discourse tasks in post-stroke aphasia. Scatterplots illustrating the correlations of word retrieval measured using NDW (left), and content word fluency measured using CIU (right) between simple picture description (y-axis) and naturalistic storytelling narrative (x-axis). Asterisks indicate significant correlations (p < 0.001).

NDW (lexical retrieval measure) derived from the simple picture description task and the naturalistic storytelling task were significantly and strongly correlated (*r* = 0.79) (Figure 2). A comparison (Fisher *r*-to-*z* transformation) between this correlation with the ones conducted between naming tests against NDW during discourse tasks indicated that measures derived from the simple picture description were a significantly better proxy of word retrieval during naturalistic discourse production compared to picture naming (all *p* ≤ 0.02).

Also, CIU (measure of content word fluency) derived from the picture description and the naturalistic storytelling tasks were significantly and strongly correlated (*r* = 0.74) (Figure 2). The strength of correlations between NDW in picture description and story-telling tasks compared to the correlation between CIU in picture description and story-telling tasks was not significantly different as indicated by Fisher r-to-z transformation. This indicated a strong relationship between each of these measures on different discourse tasks.

Results from two simultaneous multiple regression analyses once on NDW and once on CIU during storytelling narrative, using naming tests, similar measures derived from the simple picture description stimulus, and other language and cognitive tests as predictors, revealed significant regression models for both NDW (R^2^ = 0.75, F(13,32) = 7.15, *p* < 0.001), and CIU (R^2^ = 0.68, F(13,32) = 5.22, *p* < 0.001), with NDW (B = 1.24, t = 5.56, *p* < 0.001), and CIU (B = 1.65, t = 5.505 *p* < 0.001) derived from picture description appearing as the only significant variables. Even when these significant variables were removed from the models, naming tasks and all other tests remained non-significant. These results indicate that measures of word retrieval and content word fluency derived from a simple discourse task (picture description) are most strongly related to similar measures on the naturalistic discourse task (storytelling narrative), and that language/cognitive tests including naming tests do not explain any additional unique variance. As one might expect, these results imply that a simple discourse tasks do provide a more accurate indicator than picture naming, of content word fluency and word retrieval during more naturalistic forms of connected speech production. However, these measures derived from the simple discourse task still require full transcription and quantitative scoring, which is time-consuming and might be challenging for clinical applications. Thus, we considered a different approach (described below) to measure content word fluency during connected speech.

### C) A fast, efficient and accurate index of content word fluency during discourse production (Target-Checklist)

The checklist of content words produced during “Cookie Theft” picture description derived from the transcripts of neuro-typical controls forms a target of 22 words (17 words and five synonyms). In neuro-typical participants, we found that the correlation between the score on the target-checklist index and CIU extracted following full transcription and scoring of the discourse samples was extremely high *(r* = 0.90, two-tailed *p* < 0.001). When this same checklist (derived using data from the neuro-typical controls) was then applied to the PWA’s picture description samples, a similarly high correlation was found between accuracy on the target-checklist index and CIU (*r* = 0.97, two-tailed *p* < 0.001).

**Figure 3:**
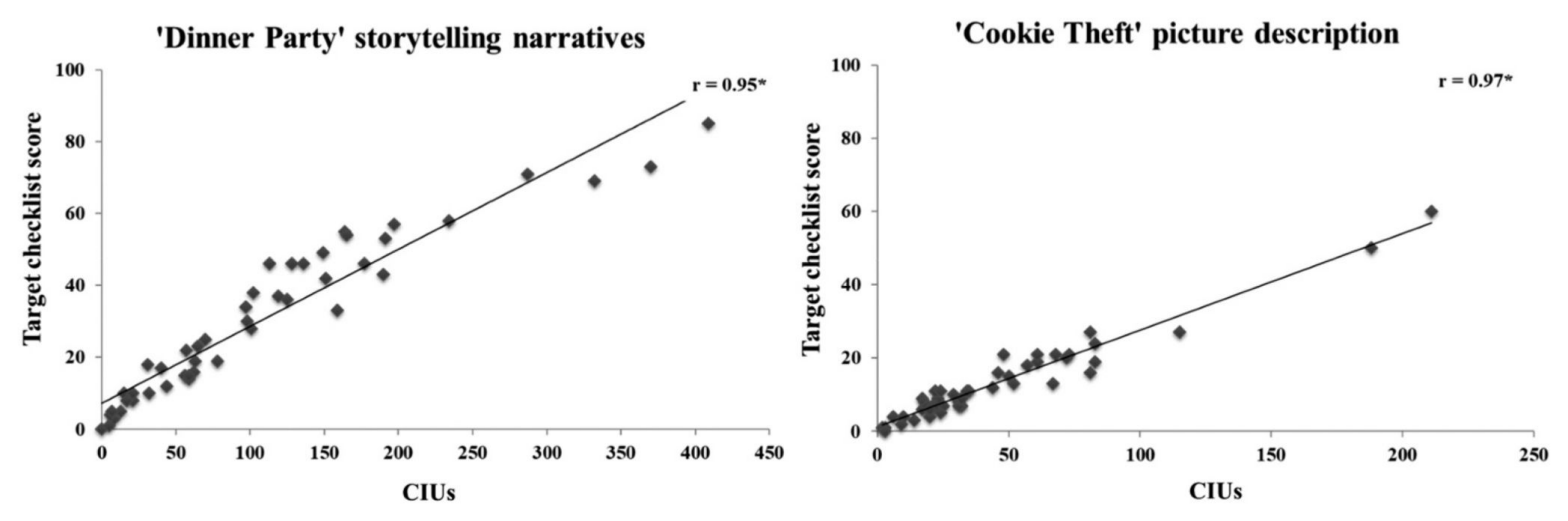
Correlations of content word fluency between scores on the new transcription-less index and CIU produced during discourse in post-stroke aphasia. Scatterplots illustrating the correlations between scores on the new transcription-less index (y-axis) and content word fluency measured using the formal transcription and quantitative scoring approach (x-axis) during storytelling narrative (left) and picture description (right). Asterisks indicate significant correlations (p < 0.001).

Thirty-three content words (25 words and eight synonyms) were extracted from the transcripts of neuro-typical controls on the “Dinner Party” storytelling narrative, and the same significantly high pattern of correlations were found in neuro-typical controls and PWA. Correlations between accuracy on the target-checklist index and CIU extracted following full transcription and scoring of discourse responses were also very high: *r* = 0.97 and *r* = 0.95, for neuro-typical controls and PWA, respectively (two-tailed *p* < 0.001). These results demonstrate high ecological validity of the new index. The correlations are illustrated in Figure 3. The target-checklists and their norms are listed in Appendix B. The scores on the target-checklists from both discourse tasks were significantly different between the groups of neuro-typical controls and PWA (*p* < 0.001), descriptive statistics are reported in Table 2.

These findings indicate that a quick and efficient transcription-less target-checklist can be derived from neuro-typical discourse samples and used as an accurate index for content word fluency in PWA. Indeed, because the relationship is so strong, it is possible to use a linear regression model to convert accuracy on the target-checklist score to the total number of CIU produced in the discourse sample: 95% of the variance of the “Cookie Theft” picture description was explained (F(1,44) = 765.15, *p* < 0.001), and 91% of the variance of the “Dinner Party” storytelling narrative was explained (F(1,44) = 447.5, *p* < 0.001).

*“Cookie Theft” picture description derived calculation for content word fluency produced by each PWA = −2.13 + (3.58 × target-checklist score)*.

*“Diner party” storytelling narrative derived calculation for content word fluency produced by each PWA = −21.06 + (4.25 × target-checklist score)*.

## Discussion

Conversations between people rely heavily on fluentconnected speech production, which depends, to a substantial extent, on the production of content words. Single-word naming and short picture description are currently utilised during clinical examination for diagnostic and therapeutic purposes. However, these measures might not reflect the person’s true ability in more naturalistic, longer discourse responses (Alyahya et al., 2020; Stark, 2019). There has been an increased interest in the use of more naturalistic forms of connected speech (such as storytelling narratives). These, however, present with challenges for clinical application, mainly because of their lengthy administration and scoring. This had led researchers to create fast and efficient approaches to analyse discourse responses. Core-lexicon is a transcription-less approach that has been recently developed to measure word retrieval (Dalton et al., 2020; Dalton & Richardson, 2015; Kim et al., 2019; MacWhinney et al., 2010). In this study, and based on the core-lexicon approach, we developed and validated a new efficient and accurate index of content word fluency during connected speech which is broadly and clinically applicable and could provide practical new measures for research and clinical trials.

We have also examined the relationship between picture naming and word retrieval during discourse production in a relatively large sample of PWA, representing a wide range of aphasia classifications and severities. The analyses revealed only weak/moderate relationship. This is consistent with the majority of the aphasiological literature, which indicated that word retrieval at single-word naming level does not correlate or predict expressive performance at connected speech discourse level (Basso et al., 1990; Bastiaanse & Jonkers, 1998; Pashek & Tompkins, 2002). Our findings of a weak-to-moderate relationship across a large and diverse sample might explain why some previous studies showed opposing results by reporting high correlations between word retrieval at single versus discourse or conversational levels (Herbert et al., 2008; Mayer & Murray, 2003). Specifically, past studies have tended to use smaller samples (≤16 participants) and thus the variation in statistical outcomes will be greater, spanning strongly significant (Herbert et al., 2008; Mayer & Murray, 2003) to non-significant correlations (Basso et al., 1990; Bastiaanse & Jonkers, 1998). In addition, correlations are only most likely to be detected if the measures are fully sampled; unlike the current study, some previous investigations have limited recruitment to mild/moderate cases only. Likewise, the current study did not impose a minimum sample length, as done previously (Fergadiotis et al., 2013; Mayer & Murray, 2003).

The current study also showed that word retrieval and content word fluency measures, specifically NDW and CIU, extracted from simple picture description strongly correlated with those measures extracted from more naturalistic forms of connected speech (story-telling narrative). The results further indicated that these measures extracted from simple picture description task, rather than other language/cognitive tests, are most strongly related to similar measures on naturalistic discourse tasks. A lack of association between cognitive tests and word fluency was found, and this could be because the discourse measures used in this study were fluency-related. Using content-related measures might yield different results, as an association between executive functions and discourse cohesive has been found in right-hemisphere mild stroke patients (Barker et al., 2017). Our findings suggest that word fluency in naturalistic discourse are mainly related to similar fluency measures on simple and short discourse task rather than other tests of general language skills (e.g., comprehension and repetition) or cognitive functions. Although this is a more positive research finding, it is important to note that even short discourse tasks are demanding and time-consuming in terms of requiring offline transcription and scoring, which reduces their clinical utility.

The transcription-less index developed in this study provides an objective measure of the overall quantity of content word production in connected speech, rather than measuring lexical retrieval in terms of NDW as done in previous studies (Dalton et al., 2020; Kim et al., 2019). Not only is this index easy to apply and time efficient but crucially scores on this new transcription-less index correlated almost perfectly with the resultant outcome from the formal lengthy transcription and quantitative scoring with regard to measures of content word fluency, demonstrating high ecological validity. Thus, this index provides a very accurate measure of the number of content words generated by each person (neuro-typical controls and PWA) during discourse production. Moreover, we derived the target-checklist independently of the targeted aphasia group (i.e., from neuro-typical controls), and demonstrated a high degree of validity across two discourse tasks in both groups. Given that the measure is both efficiently derived and highly accurate, it offers a useful approach not only for clinical application where there is much less time available with a need for an accurate clinical index but also for research purposes and as part of a core set of discourse outcome measures (Dietz & Boyle, 2018) to be utilised in clinical trials. This approach developed in this study can be administered online without the need for transcription or offline analysis, and thus allows the inclusion of more naturalistic forms of connected speech as part of the routine clinical examination. Indeed, the approach is easy to apply and does not require advanced linguistic skills, and thus the index can be used by all healthcare professionals (e.g., neurologists, psychiatrists, and nurses) to screen for word fluency post-stroke.

The scoring of the newly developed index utilises an easy and targeted objective scoring approach, which involves counting up the target words in the checklist. Therefore, it was not necessary to compute reliability. However, the reliability of this approach can be tackled in future discourse studies along with computing the reliability of the other discourse measures used in this study. Since the target-checklist is stimulus dependent, future studies can also derive and validate this approach to other discourse stimuli, preferably on a larger dataset, such as the AphasiaBank (MacWhinney et al., 2011). Several discourse measures have been recently incorporated into automated discourse analyses programs, such as the Systematic Analysis of Language Transcripts (SALT) software, and the Computerized Language Analysis (CLAN) program. Future work could aim at incorporating and instantiating the content word fluency index into these programs. It must be acknowledged that the focus of this study was content word production in connected speech, and other discourse measures, such as syntactic complexity, coherence and content richness, were beyond the scope of the current study. These measures, however, provide valuable theoretical and clinical implications and should be addressed in other discourse studies.

## Conclusions

The findings from the current study endorse the incorporation of naturalistic discourse tasks into the aphasiological clinical examination. This can be achieved through a novel time-efficient and sensitive index for measuring content word fluency during connected speech using a target-checklist. This approach allows clinicians to collect and score the discourse samples online during clinical examinations or research testing without the need for offline transcription and scoring. This meets the goal of “transcription-less discourse sampling” that has been previously described as “a clinician’s dream” (Armstrong et al., 2007).

## Supplementary Material

Appendices

## Figures and Tables

**Table 1:** Participant’s demographic information.

Demographic variables	Neuro-typical adult group (N = 20)	Aphasia group (N = 46)
**Sex**: Male: female ratio	9:11	32:14
**Age**: Mean (range, SD)	68.85 (57–84, 8.47)	63.21 (44–87, 11.93)
**Education**: Mean (range, SD)	14 (9–19, 2.8)	12.65 (9–19, 2.59)
**Time-post stroke onset**: Mean months (range, SD)	N/A	69.43 (16–280, 48.86)
BDAE* **aphasia classification:**	N/A	Fluent aphasia: Anomia = 20 Conduction = 4 Transcortical Sensory = 1 Non-fluent aphasia: Transcortical Mixed = 1 Broca’s = 9 Mixed non-fluent = 8 Global = 3

* BDAE = Boston Diagnostic Aphasia Examination (Goodglass & Kaplan, 1983).

**Table 2:** Descriptive statistics of the measures extracted from the discourse samples produced by the neuro-typical and aphasia groups.

Discourse	Measure	Neuro-typical group			Aphasia group		
		Mean	SD	Range	Mean	SD	Range
Storytelling narrative	-Length (number of tokens)	265.3	140.41	101–706	156.7	114.53	8–454
	-Duration (seconds)	128.58	64.64	43–322	190.2	109.78	42–620
	-CIU	253.85	136.86	99–672	109.2	96.78	0–409
	-NDW	99.90	32.41	55–184	51.37	35.03	0–140
	-Target-checklist*	63.65	32.75	26–165	30.6	21.94	0–85
Picture description	-Length (number of tokens)	107.65	49.14	56–252	66.57	59.70	6–315
	-Duration (seconds)	49.32	26.02	27–118	95.09	66.22	11–310
	-CIU	102.95	47.81	53–243	45.26	41.9	2–211
	-NDW	57.85	18.08	32–117	27.91	19.39	2–90
	-Target-checklist*	27.5	10.49	15 – 55	13.22	11.48	0–60

All measures were significantly different between the two groups across both discourse tasks (*p* < 0.01).

* The index measure developed in this study.
