# Supplementary material for: An efficient, accurate and clinically-applicable index of content word fluency in Aphasia
Source: Aphasiology. Author manuscript; Available in PMC 2023 Feb 3. (PMC7613208; doi:10.1080/02687038.2021.1923946)
Supplement: Appendices [file EMS149081-supplement-Appendices.pdf]

Appendices

Appendix A: Performance of persons with aphasia on language and cognitive tests

ID	CNB	BNT	ANB	Rep	Noun PWM	Verb PWM	WPM	Sent	Ravens	Brixton	Digit- F	Digi-B
NH	93.75	88.33	85.71	100.0	98.98	100.00	98.44	90.63	94.44	70.91	62.50	57.14
Ebe	82.81	38.33	78.57	81.25	94.90	96.88	100.0	75.00	66.67	47.27	50.00	28.57
KA	84.38	61.67	77.55	96.25	95.92	93.75	100.0	78.13	77.78	65.45	87.50	42.86
PBL	34.38	15.00	36.73	38.75	94.90	96.88	100.0	71.88	97.22	80.00	37.50	0.00
DL	78.13	76.67	95.92	85.00	98.98	100.00	100.0	100	94.44	74.55	62.5	42.86
TK	15.63	08.33	45.92	35.00	85.71	96.88	100.0	87.50	69.44	54.55	37.50	42.86
MH	06.25	05.00	27.55	13.75	78.57	84.38	100.0	59.38	80.56	74.55	25.00	28.57
DCs	67.19	43.33	89.80	72.50	100.00	100.00	100.0	93.75	100.0	81.82	62.50	57.14
KS	31.25	13.33	70.41	93.75	75.51	65.63	71.88	84.38	86.11	52.73	100.0	57.14
RH	3.13	01.67	36.73	21.25	94.90	100.00	96.88	62.50	83.33	61.82	25.00	28.57
WE	78.13	55.00	71.43	71.25	95.92	93.75	100.0	84.38	91.67	70.91	62.5	42.86
Gha	87.50	78.33	79.59	91.25	96.94	100.00	95.31	93.75	83.33	69.09	50.00	42.86
DF	87.50	50.00	84.69	93.75	89.80	87.50	100.0	62.50	88.89	43.64	37.50	28.57
RL	84.38	63.33	82.65	61.25	97.96	96.88	96.88	62.50	80.56	72.73	62.50	57.14
JBr	93.75	85.00	86.73	97.50	98.98	100.00	100.0	96.88	97.22	67.27	75.00	71.43
Ebo	89.06	55.00	91.84	100.0	94.90	87.50	100.0	87.50	97.22	69.09	50.00	57.14
WC	54.69	55.00	72.45	76.25	95.92	100.00	98.44	75.00	52.78	70.91	62.50	42.86
BH	95.31	66.67	80.61	100.0	87.76	87.50	98.44	78.13	66.67	67.27	62.50	57.14
JS	89.06	43.33	69.39	90.00	97.96	100.00	100.0	81.25	100.0	65.45	50.00	57.14
AL	93.75	88.33	82.65	100.0	95.92	100.00	100.0	84.38	91.67	60.00	87.50	85.71
MB	87.50	73.33	87.76	87.50	98.98	100.00	100.0	93.75	97.22	76.36	50.00	28.57
DM	75.00	71.67	86.73	73.75	100.00	96.88	98.44	56.25	91.67	50.91	37.50	00.00
PW	04.69	05.00	11.22	00.00	83.67	90.63	100.0	56.25	50.00	34.55	25.00	00.00
JW	65.63	38.33	69.39	65.00	91.84	100.00	96.88	90.63	88.89	61.82	87.50	28.57
Mad	84.38	76.67	77.55	95.00	94.90	100.00	98.44	81.25	83.33	63.64	62.50	00.00
RR	54.69	23.33	54.08	51.25	92.86	90.63	98.44	56.25	88.89	43.64	25.00	28.57
AD	53.13	50.00	46.94	57.50	90.82	84.38	96.88	68.75	63.89	30.91	75.00	42.86
KL	4.69	01.67	19.39	06.25	77.55	87.50	92.19	28.13	88.89	61.82	00.00	00.00
GP	71.88	56.67	72.45	95.00	100.00	96.88	98.44	78.13	97.22	78.18	37.50	28.57
JSc	71.88	53.33	68.37	90.00	80.61	100.00	98.44	75.00	77.78	43.64	62.50	42.86
AG	87.50	78.33	70.41	77.50	91.84	93.75	100.0	87.50	75.00	56.36	100.0	100.0
DC	4.69	00.00	0.00	00.00	90.82	93.75	100.0	75.00	88.89	76.36	37.50	42.86
CH	84.38	60.00	77.55	92.50	94.90	100.00	100.0	84.38	91.67	76.36	50.00	28.57
AS	06.25	01.67	0.00	36.25	56.12	59.38	76.56	50.00	47.22	47.27	25.00	0.00
MD	46.88	38.33	8.16	50.00	71.43	46.88	96.88	12.50	38.89	58.18	37.50	28.57
AB	76.56	41.67	26.53	86.25	92.86	90.63	95.31	75.00	88.89	88.89	37.50	28.57
PR	60.94	38.33	74.49	85.00	88.78	93.75	100.0	87.50	80.56	50.91	75.00	0.00
GL	68.75	31.67	50.00	100.0	95.92	96.88	96.88	65.63	91.67	58.18	37.50	28.57
DB	07.81	08.33	23.47	85.00	74.49	75.00	64.06	31.25	86.11	40.00	37.50	28.57
JM	00.00	00.00	0.00	01.25	72.45	75.00	78.13	46.88	91.67	91.67	25.00	0.00
DR	14.06	03.33	6.12	88.75	73.47	84.38	62.50	46.88	83.33	65.45	37.50	0.00
Gho	25.00	16.67	81.22	62.50	64.29	46.88	85.94	43.75	61.11	34.55	25.00	00.00
JB	39.06	10.00	9.18	43.75	71.43	75.00	100	34.38	77.78	60.00	75.00	42.86

(*Continued*)

(Continued).

ID	CNB	BNT	ANB	Rep	Noun PWM	Verb PWM	WPM	Sent	Ravens	Brixton	Digit- F	Digi-B
PM	59.38	51.67	54.08	65.00	84.69	90.63	92.19	62.50	47.22	30.91	25.00	28.57
CF	43.75	20.00	29.59	70.00	84.69	84.38	89.06	68.75	91.67	50.91	25.00	00.00
DBb	00.00	00.00	0.00	37.50	62.24	68.75	57.81	12.50	30.56	38.18	25.00	00.00

Scores are given as percentages.

Abbreviations: CNB = Cambridge Naming Battery (Bozeat et al., 2000); BNT = Boston Naming Test (Kaplan et al., 1983); ANB = action pictures from the Object and Action naming Battery (Druks & Masterson, 2000); Rep = immediate word repetition from the Psycholinguistic Assessments of Language Processing in Aphasia (Kay et al., 1992); Noun PWM = Noun picture-to-word matching (Alyahya et al., 2018b); Verb PWM = Verb picture-to-word matching (Alyahya et al., 2018a); WPM = spoken word-to-picture matching test from the Cambridge Semantic Battery (Bozeat et al., 2000); Sent = spoken sentence comprehension subtest from the Comprehensive Aphasia Test (Swinburn et al., 2005); Raven = Raven's Coloured Progressive Matrices (Raven, 1962); Brixton = Brixton Spatial Rule Anticipation Task (Burgess & Shallice, 1997); Digit F = forward digit span (Wechsler, 1987); Digit B = backward digit span (Wechsler, 1987).

Appendix B: Target checklists used in the novel content word fluency index

Target words	Acceptable Inflections	Acceptable synonyms and their inflections
"Dinner Party" Storytelling Narrative (Fletcher & Birt, 1983)		
Man		Husband, Chap
Is	Was	
Phone	Phones, Phoning	Ring, Rings, Ringing
Dinner	Dinners	
Prepare	Prepares, Preparing, Prepared,	
Meal	Meals	Food, Foods
Lady		Wife, Women
Cook	Cooks, Cooking, Cooked	
Wash	Washes, Washing	
Put	Puts, Putting	
Fish	Fishes	
Table	Tables	
Candle	Candles	
Cat	Cats	
Get	Gets, Getting, Gotten	
Dress	Dresses, Dressing, Dressed	
Are	Were	
Had		
Come	Comes, Coming, Came	Arrive, Arrives, Arriving, Arrived
Have	Has	
Look	Looks, Looking, Looked	
Go	Goes, Going, Gone	
Run	Runs, Running, Ran	Dash, Dashes, Dashing, Dashed
Chip	Chips	
Shop	Shops	
**"Cookie Theft" picture description** (Goodglass & Kaplan, 1983)		
Mother		Mum, Lady, Woman
Is	Was	
Look	Looks, Looking, Looked	
Wash	Washes, Washing, Washed	
Sink	Sinks	
Overflow	Overflows, Overflowing, Overflowed	
Water		
Floor		
Children		Kids
Boy		
Stool		
Go	Goes, Going, Gone	
Fall	Falls, Falling	
Get	Gets, Getting, Got	
Cookie	Cookies	Biscuit, Biscuits
Jar		
Girl		
